# Adding unaligned sequences into an existing alignment using MAFFT and LAST

**DOI:** 10.1093/bioinformatics/bts578

**Published:** 2012-09-27

**Authors:** Kazutaka Katoh, Martin C. Frith

**Affiliations:** ^1^Laboratory of Systems Immunology, Immunology Frontier Research Center (IFReC), Osaka University, Yamadaoka, Suita 565-0871 and ^2^Computational Biology Research Center, National Institute of Advanced Industrial Science and Technology (AIST), Aomi, Koto-ku, Tokyo 135-0064, Japan

## Abstract

Two methods to add unaligned sequences into an existing multiple sequence alignment have been implemented as the ‘–add’ and ‘–addfragments’ options in the MAFFT package. The former option is a basic one and applicable only to full-length sequences, whereas the latter option is applicable even when the unaligned sequences are short and fragmentary. These methods internally infer the phylogenetic relationship among the sequences in the existing alignment and the phylogenetic positions of unaligned sequences. Benchmarks based on two independent simulations consistently suggest that the “–addfragments” option outperforms recent methods, PaPaRa and PAGAN, in accuracy for difficult problems and that these three methods appropriately handle easy problems.

**Availability:** http://mafft.cbrc.jp/alignment/software/

**Contact:** katoh@ifrec.osaka-u.ac.jp

**Supplementary information:** Supplementary data are available at *Bioinformatics* online

Recent advances of sequencing technologies produce a large amount of sequence data. A relatively small number of sequences are carefully aligned and annotated in databases, for example, Cole *et al.* (2009); Punta *et al.* (2012); Sigrist *et al.* (2010). Sometimes we have to align newly determined sequences into an existing multiple-sequence alignment (MSA) taken from such databases. This is more efficient than the entire rebuilding of an MSA from a set of ungapped sequences. Moreover, the resulting alignment retains biological knowledge that was incorporated into the existing alignment. Most likely, for such analyses, we should not apply profile–profile or profile–sequence alignment, which converts an existing alignment to a profile and then aligns it with new sequences. If this is applied, the phylogenetic relationship between the new sequences and the sequences in the existing alignment cannot be considered, and thus, the alignment quality is low. Recently, several tools (Berger and Stamatakis, 2011; Löytynoja *et al.*, 2012; Sun and Buhler, 2012) that can align new sequences to an existing MSA have been developed, especially for short DNA reads.

MAFFT (Katoh *et al.*, 2002) is a general purpose MSA program, and now it supports the feature to add sequences into an existing alignment, considering the phylogenetic relationship. This article describes its two options, –add and –addfragments, which are different combinations of existing routines in the MAFFT package and a rapid local alignment method, LAST (Kiełbasa *et al.*, 2011). The –add option is simpler but has a limitation in handling short sequences. The –addfragments option is specially designed for short sequences. Here, we focus on situations with short unaligned DNA sequences because the needs for aligning such data are recently increasing.

The –add option adopts a standard progressive alignment method (Feng and Doolittle, 1987; Higgins and Sharp, 1988) to build an alignment using a guide tree, except for nodes where the existing alignment should be preserved. The calculation procedure is:

1. Let the number of sequences in the existing alignment be *N* and the number of new sequences be *n*.
2. Compute a distance matrix, with (*N* + *n*)^2^ elements, of the sequences in the existing alignment and the new sequences. The distance can be computed by several different methods, including that based on the local pairwise alignment scores by dynamic programming (DP) [indicated as –add (DP) in Tables below] and that based on the number of shared 6mers [indicated as –add (6mer)]. Global alignment can also be selected here, but we did not test this option. In all cases, a similarity score is converted to a distance as in Katoh *et al.* (2002).
3. Build a guide tree with (*N* + *n*) tips from the distance matrix.
4. Build an alignment using the guide tree. For each node,
  - If all the children of the node are in the existing alignment or already aligned to the existing alignment, then no alignment calculation is necessary.
  - If a new sequence is involved in the node and the sequence is not yet aligned in the previous steps, alignment calculation is performed. If new gaps are introduced here, gaps are also inserted into the existing alignment as necessary.

This option can be used for adding full-length sequences into an existing alignment. However, when new sequences to be added are fragments that do not overlap each other, the distances among them are mostly meaningless because non-homologous fragments are forced to be aligned.

For handling short unaligned sequences, another option, –addfragments, has been designed. In this option, new sequences are independently added into an alignment, without considering the relationship among new sequences. This strategy was adopted because most short sequences are not expected to overlap each other. The calculation procedure is as follows:

1. Compute a distance matrix of the existing alignment consisting of *N* sequences. The pairwise alignment scores are converted into pairwise distances (between 0 and 1) as in Katoh *et al.* (2002).
2. Compute *N* × *n* pairwise local alignments between the *N* sequences in the existing alignment and the *n* new sequences. Only the best alignment is used per sequence pair. If no alignment is found, the distance is set to 1. By default, the Smith–Waterman algorithm (Smith and Waterman, 1981) is used [indicated as –addfragments (DP) in Tables below]. It can be replaced by LAST for faster computation [indicated as –addfragments (LAST)] for nucleotide data.
3. For each of the *n* new sequences, the same calculation as in the –add option is repeated independently. That is,
  - Build a guide tree consisting of *N* + 1 tips, using the distances calculated in steps 1 and 2.
  - Compute a multiple alignment consisting of *N* + 1 sequences using the guide tree computed at the previous step.
4. Combine *n* different MSAs, calculated in step 3, into one MSA, inserting gaps as necessary. For each of the *n* MSAs,
  - If no gap was inserted into the existing alignment in step 3, then no new gap is necessary in this step.
  - If gaps were inserted into the existing alignment to align the new sequence in step 3, then corresponding gaps are inserted in the other new sequences in this step.

If different new sequences create gaps in the same position in the existing alignment, then the inserted new sequences are aligned to each other.

Each of steps 1–3 runs in parallel, on a multi-core PC.

We implemented the aforementioned options in MAFFT version 6.927. To test their performance, we used the same datasets as those used in the PAGAN article (Löytynoja *et al.*, 2012). These are two independently simulated datasets, prepared by Löytynoja *et al.* (2012) and Mirarab *et al.* (2012). We included two representative methods, PaPaRa version 2.0 and PAGAN version 0.38, in the comparison because they can be used in situations similar to ours.

Table 1 shows the result for one of the two datasets. See Supplementary Material for details on the benchmark settings and results. The –addfragments option generally outperformed the other methods in accuracy, except for easy problems. This table also shows results of profile–sequence alignment. An existing alignment was converted to a profile, and each new sequence was separately aligned to the profile using mafft–profile. The application of profile alignment in this situation is conceptually wrong, as noted at the beginning. As shown in Supplementary Table S1, the –add option worked well for full-length sequences, although it could not appropriately handle fragmentary sequences, as expected.

**Table 1.** Comparison using SEPP dataset

Method\data	1000M2	1000M2-p	1000M3	1000M3-p	1000M4	CPU time (s)	Actual time on 8 cores (s)
MAFFT							
–addfragments (DP)	0.8313	0.9970	0.9299	0.9975	0.9986	7487	1 026
–addfragments (LAST)	0.8303	0.9970	0.9288	0.9974	0.9986	2635	676.7
–add (DP)	0.7280	0.9755	0.8440	0.9744	0.9790	15 360	2 160
–add (6mer)	0.2716	0.5935	0.4163	0.6398	0.8450	135.8	
MAFFT–profile	0.0345	0.0395	0.1077	0.1241	0.5991		
PaPaRa	0.6739	0.9653	0.8339	0.9764	0.9973	2601	375.8
PAGAN (fast option)		0.9292		0.9316	0.9857	376.5	

The estimated alignments were compared with the true alignments to measure the accuracy (the number of correctly aligned letters/the number of aligned letters in the true alignment). The accuracy scores were averaged for all the pairs and then averaged for all the 20 simulated replicates. See Supplementary Material for the details.

The –addfragments option performed well in comparison with existing methods that were specially designed for aligning short sequences. For the SEPP dataset, as shown in Table 1, it generally outperformed PaPaRa and PAGAN, but in the easiest case (1000M4), the difference was minor. For the PAGAN dataset (see Supplementary Table S1), the difference among these three methods was small, but a slight tendency was observed that PAGAN performed better for easy cases and that –addfragments performed better for difficult cases.

Our approach differs from PaPaRa and PAGAN, in that it internally infers a tree of the existing alignment and the phylogenetic positions of new sequences in the tree. Thus, it can be applied even when we have no sound phylogenetic information of the existing alignment. This feature can be a disadvantage, when the number of sequences, *N*, in the existing alignment is large. Our method computes and stores *O*(*N*^2^) distances, whereas tree information needs only *O*(*N*) space. We are planning to try more approximate methods to overcome this limitation in the future. On the other hand, the –addfragments option is scalable to the number of new sequences, *n*.

We had to use simulation because there is no established benchmark dataset based on actual sequences for this purpose. Thus, the assessment here is still insufficient, especially for comparing methods with different scoring systems. We cannot completely exclude the possibility that the observations just reflect the fitness of the parameter set to the simulation setting. However, we can safely compare different MAFFT options that use the same parameter set. They behaved similarly for the two independent datasets, generated by different programs, INDElible and ROSE. Thus, it is unlikely that the results are artificial because of simulation settings. In the future, benchmark datasets of actual sequences should be established for this type of problem.

We did not assess the accuracy of phylogenetic placement of the fragmentary sequences. As noted previously, MAFFT does not use a user-defined tree of the existing alignment, unlike PaPaRa and PAGAN. MAFFT calculates the phylogenetic positions of new sequences in an internally constructed tree, instead of a user-defined tree. Thus, it is difficult to make straightforward assessments about the correctness of the phylogenetic position in a given tree. Tests of phylogenetic placement using alignments by different methods, including that described here, are being performed by Tandy Warnow’s group (personal communication).

The present results suggest the usefulness of a simple and natural algorithm for adding new sequences into an alignment. The straightforward strategy implemented as mafft –addfragments showed high performance for problems that were previously used for assessing this type of method. It can be a basis for extensions in several directions, such as specific support for NGS data, and faster and more scalable calculation.

## Supplementary Material

Supplementary Data
