# Supplementary material for: Adding unaligned sequences into an existing alignment using MAFFT and LAST
Source: Bioinformatics. 2012 Sep 27;28(23):3144–6. doi: 10.1093/bioinformatics/bts578 (PMC3516148; doi:10.1093/bioinformatics/bts578)
Supplement: Supplementary Data [file supp_28_23_3144__index.html]

Adding unaligned sequences into an existing alignment using MAFFT and LAST — Adding unaligned sequences into an existing alignment using MAFFT and LAST — Supplementary Data 

# Adding unaligned sequences into an existing alignment using MAFFT and LAST

## Supplementary Data

files

**Files in this Data Supplement:**

- Supplementary Data - pdf file
