# Supplementary material for: Adding unaligned sequences into an existing alignment using MAFFT and LAST
Source: Bioinformatics. 2012 Sep 27;28(23):3144–6. doi: 10.1093/bioinformatics/bts578 (PMC3516148; doi:10.1093/bioinformatics/bts578)
Supplement: Supplementary Data [file supp_bts578_suppl.pdf]

Supplementary information

We tested the performance of the `--add` and `--addfragments` options using two independently simulated datasets, the PAGAN dataset and the SEPP dataset. In addition to the options described in the main text, we also tested several additional variants, in which distance is computed by different methods, LAST (nucleotide only), 10mer (nucleotide only), 6mer and DP.

The PAGAN dataset was provided by Ari Löytynoja. We selected four cases (model trees (EnsTr1, EnsTr3) × length of unaligned sequences (30, full length)) out of the original 12 cases, such that the variety of conditions is kept. EnsTr1 and EnsTr3 correspond to relatively conserved and diverged sequences, respectively. Hereafter these cases are refered to as EnsTr1-30, EsnTr3-full, EnsTr1-30 and EnsTr1-full. Each instance consists of an MSA of 67 sequences and unaligned sequences, generated by INDElible [1]. The unaligned sequences were added into the MSA of 67 sequences (no re-alignment was performed for this MSA), by the methods to be tested. They originally used the simNGS program to simulate Illumina sequencing errors. However, because its effect is small (Löytynoja, personal communication), here we directly used the sequence data generated by INDElible, without additional errors. We confirmed that this data largely reproduces the results reported in their paper. This suggests that the effect of Illumina sequencing error is small in comparison to that of evolutionary divergence. We tested mainly the nucleotide data, but we also tested amino acid data as far as supported.

The SEPP dataset was taken from [2]. It consists of the 1000M2, 1000M3 and 1000M4 subsets, which correspond to hard, medium and easy problems, respectively. Each instance has 500 aligned sequences and 5,000 fragmentary sequences, generated by the ROSE program [3]. The original aim of this dataset is to assess the accuracy of inference of phylogenetic placement of new sequences, rather than alignment accuracy. However, here we focus on alignment accuracy. Since the problem size is large, we applied a fast option of PAGAN, according to their paper. We also tested a slower option (same as that used for the PAGAN dataset), but observed no improvement in the accuracy. Since PAGAN aligns only some of the fragments when the input sequences are diverged, we re-calculated the accuracy score of the other methods using only the sequences aligned by PAGAN, as necessary (referred to as 1000M2-p and 1000M3-p).

Our test was performed on a Linux PC with 4 × Quad-Core AMD Opteron and 64GB of RAM. There is another PaPaRa package that supports GPGPU for rapid calculation [4], but here we did not use it. PaPaRa and PAGAN used the true model tree of the existing alignment, while MAFFT used trees that are internally inferred from the given alignment, as described above. This might make the

comparison biased toward PaPaRa and PAGAN, because they use information that is not available in actual analyses. However, the effect of the use of the model tree is reported to be negligible [5]. The phylogenetic positions of the new sequences were not provided to any of the methods.

References

1. Fletcher W, Yang Z. INDELible: a flexible simulator of biological sequence evolution. *Mol Biol Evol,* 2009; **26**:1879–1888.

2. Mirarab S, Nguyen N, Warnow T. SEPP: SATé-Enabled phylogenetic placement. *Pac Symp Biocomput,* 2012; **17**:247–258.

3. Stoye J, Evers D, Meyer F. Rose: generating sequence families. *Bioinformatics,* 1998; **14**:157–163.

4. Alachiotis NC, Berger SA, Stamatakis A. Coupling SIMD and SIMT architectures to boost performance of a phylogeny-aware alignment kernel. *BMC Bioinformatics,* 2012; **13**:196.

5. Löytynoja A, Vilella AJ, Goldman N. Accurate extension of multiple sequence alignments using a phylogeny-aware graph algorithm. *Bioinformatics,* 2012; **28**:1684–1691.

Table 1: Comparison using PAGAN dataset

A. DNA

Method \ Data	EnsTr3-30	EnsTr3-full	EnsTr1-30	EnsTr1-full	CPU time[†] (sec.)	Actual time[‡] (sec.)
MAFFT						
`--addfragments` (DP)[1]	0.9181	0.9615	0.9632	0.9847	16.72	3.823
`--addfragments` (LAST)[2]	0.9167	0.9479	0.9595	0.9800	7.199	3.812
`--addfragments` (10mer)[3]	0.8495	0.9514	0.9439	0.9802	3.737	1.951
`--addfragments` (6mer)[4]	0.8184	0.9486	0.9361	0.9806	1.689	1.406
`--add` (DP)[5]	0.6502	0.9603	0.7813	0.9837	102.1	17.67
`--add` (LAST)[6]	0.7181	0.9460	0.8143	0.9765	45.75	12.13
`--add` (10mer)[7]	0.4165	0.9494	0.4531	0.9793	5.189	
`--add` (6mer)[8]	0.4525	0.9470	0.4732	0.9805	3.109	
PaPaRa[9]	0.9135	0.9385	0.9623	0.9782	6.293	2.047
PAGAN[10]	0.8383	0.9817	0.9572	0.9932	817.7	

B. Protein

Method \ Data	EnsTr3-30	EnsTr3-full	EnsTr1-30	EnsTr1-full	CPU time[†] (sec.)	Actual time[‡] (sec.)
MAFFT						
`--addfragments` (DP)[1]	0.9449	0.9618	0.9757	0.9849	7.045	2.169
`--addfragments` (6mer)[4]	0.8829	0.9485	0.9521	0.9850	1.534	1.224
`--add` (DP)[5]	0.8328	0.9653	0.8699	0.9845	15.00	3.539
`--add` (6mer)[8]	0.4973	0.9474	0.4766	0.9801	0.7266	
PAGAN[10]	0.9378	0.9857	0.9785	0.9946	182.2	

The alignment between each new sequence and its closest relative in the existing alignment was used to measure the alignment accuracy (the number of correctly aligned letters / the number of aligned letters in true alignment). The accuracy scores were averaged for all the pairs and then averaged for all the 50 simulated replicates.

[1–6] Command-line arguments for each program are:

[1] `mafft --addfragments N A`

[2] `mafft --addfragments N --lastmultipair A`

[3] `mafft --addfragments N --10merpair A`

[4] `mafft --addfragments N --6merpair A`

[5] `mafft --add N --localpair A`

[6] `mafft --add N --lastpair A`

[7] `mafft --add N --10merpair A`

[8] `mafft --add N --6merpair A`

[9] `papara -t T -s A -q N`

[10] `pagan --ref-treefile T --ref-seqfile A --queryfile N --fast-placement --test-every-internal-node --exhaustive-placement`

where `N` is new sequences; `A` is existing alignment; `T` is tree of existing alignment

[†] Average CPU time for completing the calculation of one instance from the EnsTr3-30 dataset.

[‡] Wall-clock time with 8 cores. `--thread 8` for mafft; `-j 8` for PaPaRa; The parallelization efficiency of the LAST options is low due to overhead to run the external processes, when the data size is small.

Table 2: Comparison using SEPP dataset

Method \ Data	1000M2	1000M2-p*	1000M3	1000M3-p*	1000M4	CPU time† (sec.)	Actual time‡ (sec.)
MAFFT							
`--addfragments` (DP)[1]	0.8313	0.9970	0.9299	0.9975	0.9986	7,487	1,026
`--addfragments` (LAST)[2]	0.8303	0.9970	0.9288	0.9974	0.9986	2,635	676.7
`--addfragments` (10mer)[3]	0.3551	0.8436	0.4912	0.8467	0.9884	511.7	262.0
`--addfragments` (6mer)[4]	0.3131	0.6852	0.4735	0.7309	0.9796	283.7	190.1
`--add` (DP)[5]	0.7280	0.9755	0.8440	0.9744	0.9790	15,360	2,160
`--add` (LAST)[6]	0.7263	0.9767	0.8473	0.9759	0.9770	8,061	1,787
`--add` (10mer)[7]	0.3309	0.6978	0.4715	0.7162	0.8196	375.0	
`--add` (6mer)[8]	0.2716	0.5935	0.4163	0.6398	0.8450	135.8	
`mafft-profile`	0.0345	0.0395	0.1077	0.1241	0.5991		
PaPaRa[9]	0.6739	0.9653	0.8339	0.9764	0.9973	2,601	375.8
PAGAN (fast)[10]	*	0.9292	*	0.9316	0.9857§	376.5	
Muscle profile	0.0280	0.0321	0.0719	0.0830	0.4863		

The alignment between each new sequence and each sequence in the existing alignment was used to measure the alignment accuracy (the number of correctly aligned letters / the number of aligned letters in the true alignment). The accuracy scores were averaged for all the pairs and then averaged for all the 20 simulated replicates.

[1–9] Same as Table 1.

[10] `pagan --ref-treefile T --ref-seqfile A --queryfile N --very-fast-placement --test-every-node`

* PAGAN aligned only a small number of fragments (1,259-2,406 out of 5,000 for 1000M2; 1,932-3,001 out of 5,000 for 1000M3). The results for each method without those skipped fragments are shown in the 1000M2-p and 1000M3-p columns.

§ For 1000M4, the number of fragments skipped by PAGAN was small (24-247 out of 5,000). So the average score for PAGAN was calculated just excluding the skipped fragments.

† Average CPU time for completing the calculation of one instance from the 1000M2 dataset.

‡ Wall-clock time with 8 cores.
